# Penetrance of the V203I variant of the PRNP gene: report of a patient with stroke-like onset of Creutzfeld-Jacob Disease and review of published cases

**DOI:** 10.1080/19336896.2022.2035479

**Published:** 2022-02-15

**Authors:** Ilaria Gandoglia, Laura Strada, Anna Poleggi, Antonio Castaldi, Massimo Del Sette, Emilio Di Maria

**Affiliations:** a Unit of Neurology, Galliera Hospital, Genoa, Italy; b Department of Neuroscience, Istituto Superiore di Sanità, Rome, Italy; c Unit of Diagnostic and Interventional Neuroradiology, Galliera Hospital, Genoa, Italy; d Unit of Neurology, IRCCS Policlinico San Martino, Genoa, Italy; e Department of Health Sciences, University of Genova, Genoa, Italy; f Unit of Medical Genetics, Galliera Hospital, Genoa, Italy

**Keywords:** *PRNP*, prion disease, stroke-like onset, genotype, penetrance, risk factor, review

## Abstract

Creutzfeldt-Jakob disease (CJD) is usually sporadic, but 10–15% of cases are caused by autosomal-dominant pathogenic variants in the prion protein gene (*PRNP*). A few *PRNP* variants show low penetrance. We report the case of a 64-year-old man, admitted to the ward with acute onset of aphasia; death occurred 6 weeks later. Brain MRI, EEG pattern and brain pathology were consistent with CJD diagnosis. Genetic analysis revealed a heterozygous V203I variant. We summarized the key clinical findings in patients carrying the V203I variant who were described to date. We also discuss the hypothesis as to whether V203I is a risk factor for CJD rather than a Mendelian disease-associated variant, as well as the possible implications of such hypothesis in the clinical scenario.

## Introduction

Prion protein (PrP) exists in multiple isoforms, predominantly the cellular, non-infectious form (PrP^C^), and the misfolded isoform, known as scrapie (PrP^Sc^). PrP^Sc^ causes prion diseases, a group of rapidly progressive neurodegenerative disorders. There are three major groups of human prion diseases: sporadic, acquired, and genetic. They include Creutzfeldt-Jakob disease (CJD), whose incidence is one-two cases per million per year [1]. Sporadic CJD (sCJD) is the most common form representing the 85% of total CJD cases. It occurs generally in late middle age with short survival post-diagnosis. Acquired forms of CJD include Kuru, iatrogenic CJD (iCJD), and variant CJD (vCJD). Genetic forms of CJD (gCJD) are associated with pathogenic mutations in the prion protein gene *PRNP* and include familial CJD, fatal familial insomnia, and Gerstmann-Schäussler-Scheinker syndrome, accounting for about 15% of cases world-wide [1]. More than 40 *PRNP* variants were associated with gCJD. Most are 100% penetrant, but some of them have very low penetrance (<1%) and might just be risk factors [2].

The c.656 G > A – p.Val203Ile variant of the *PRNP* gene (V203I, position 20:4680473 according to assembly GRCh37) was first described in a 69-year-old Italian patient with CJD [3]. Subsequently, additional patients of different ancestries were described, namely from Korea [4], China [5,6], Japan [7] (the patient was homozygous for the V203I variant) and Italy [8]. Kovacs and co-workers focussed on tau pathology in a case series including one Austrian and two French patients [9]. The penetrance of the V203I variant was quantified in a large multicentric study, which collected 16 carriers (17 alleles). Based on the frequency of V203I in CJD patients as compared with allelic frequencies in large control cohorts, and the lack of segregation in families, the study concluded that V203I is unlikely to be a highly penetrant Mendelian disease variant and could be benign or associated with an increased prion disease risk [2].

We report the case of a carrier of the *PRNP* V203I variant diagnosed with CJD, who showed stroke-like acute onset and rapid course of illness. We reviewed the published cases of patients carrying the *PRNP* V203I variant and discuss the possible correlation between the V203I genotype and CJD development.

## Case presentation

**Figure 1.**
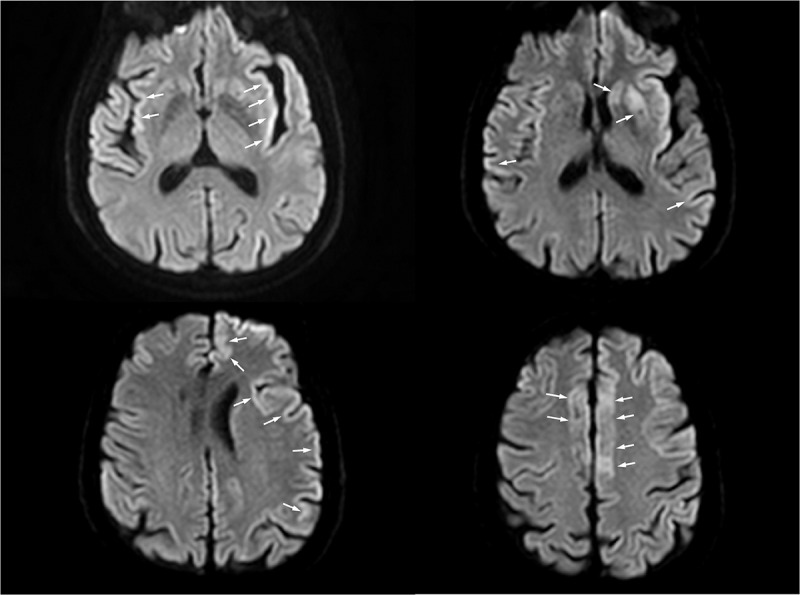
The MRI axial DWI sequence shows signal hyperintensity involving the right frontal, the left frontal and parietal cortical areas (known as cortical ribbon sign); insular cortex (especially on the left) and left corpus striatum are also involved. Areas of interest are highlighted by arrows.

The patient was a 64-year-old man with an unremarkable clinical history and a normal social and working life until symptoms appeared. No family history of neurological diseases was reported. The patient was admitted to the Neurological ward with word-finding difficulties that suddenly occurred 4 days before admission. After 3 days of hospitalization, the patient showed a subacute onset of involuntary chewing movements. The brain magnetic resonance imaging (MRI) displayed no abnormality. Few days later (approximately two weeks after admission), the clinical general conditions rapidly worsened with deterioration of consciousness, rapidly progressing into coma. A second brain MRI (9 days after the first MRI) demonstrated bilateral hyperintensity of basal ganglia, insula and bilateral cortex with left temporal lobe cortical ribboning on diffusion weighted imaging (DWI), that is a pattern consistent with CJD diagnosis (Figure 1). This hypothesis was further explored and eventually supported by increased levels of 14.3.3 and tau proteins in cerebrospinal fluid and by the typical EEG pattern characterized by pseudoperiodic generalized triphasic slow waves (not shown). The patient died 6 weeks after the admission. Autopsy confirmed the CJD diagnosis. Sanger sequencing of the *PRNP* gene was performed by using current methods [10]. The analysis revealed the heterozygous V203I genotype (c.656 G > A – p.Val203Ile) and the homozygous Met/Met genotype at codon 129. The family had provided the appropriate consent to the clinical study, including the relevant genetic analyses.

## Discussion

After an extensive literature search on published cases with CJD and the V203I genotype, seven articles reporting clinical features were included for data extraction. A summary of the findings is reported in Table 1.

**Table 1.** Clinical features of CJD patients carrying the *PRNP* V301I variant (nr = not reported)

Publication	Peoc’h 2000 [3]	Jeong 2010 [4]	Shi 2013 [5]	Komatsu 2014 [7]	Cistaro 2017 [8]	Kovacs 2017 [9]	Kovacs 2017 [9]	Kovacs 2017 [9]	Tang 2018 [6]	Present case
V301I genotype	Val/Ile	Val/Ile	Val/Ile	Ile/Ile	Val/Ile	Val/Ile	Val/Ile	Val/Ile	Val/Ile	Val/Ile
M129V genotype	Met/Met	Met/Val	Met/Met	Met/Met	nr	Met/Met	Met/Met	Met/Met	Met/Met	Met/Met
Sex	M	F	M	F	F	M	F	M	F	M
Family history	negative	nr	negative	negative	negative	nr	nr	nr	nr	negative
Age at diagnosis	69	66	80	73	48	76	71	69	61	64
Duration of disease	35 days	2 months	2 months	24 months	nr	7 months	7 months	2 months	7 months	6 weeks
Cognitive symptoms	yes	yes	yes	yes	yes	yes	yes	yes	yes	yes
Behavioural symptoms	yes	yes	no	no	nr	nr	nr	nr	yes	yes
Psychotic symptoms	yes	no	no	no	nr	yes	yes	no	no	yes
Ataxia	yes	yes	yes	no	yes	yes	no	yes	nr	yes
Tremor	yes	yes	yes	no	nr	yes	yes	yes	nr	yes
Rigidity	no	yes	yes	no	nr	yes	yes	yes	nr	yes
Brain MRI	nr	typical	typical	typical	typical	nr	typical	nr	typical	typical
EEG	typical	nr	typical	typical	typical	typical	typical	typical	moderate abnormality (not specified)	typical
CSF	nr	nr	14.3.3	14.3.3 and tau	tau	14.3.3	14.3.3	14.3.3	negative	14.3.3 and tau
Pathology	nr	typical	nr	typical	nr	prominent astrocytic tau pathology	prominent astrocytic tau pathology	prominent astrocytic tau pathology	nr	typical

The most peculiar clinical feature of the present case was the stroke-like onset, which is a rare clinical presentation representing 2% of all cases [11]. To date, stroke-like onset has not been related to any specific genetic variant of *PRNP*. The clinical course in our patient was very rapid, as compared to most patients carrying the V203I variant. However, the duration of illness was highly variable, spanning from 5 weeks to 24 months after the disease onset (Table 1). Age at onset varied from 48 to 80 years (mean 67.7, SD 8.9). All patients presented with cognitive deterioration, variably associated with behavioural and psychiatric symptoms. Signs of movement disorder were present in all cases, with variable association with ataxia, tremor, and rigidity. Of note, family history was unremarkable in our patient and in four patients for whom family history was recorded (Table 1). The patient homozygous for the V203I variant [7] did not show peculiar features as compared to the heterozygous cases.

In summary, all cases carrying one or two variant alleles showed rather typical neurological signs, cerebrospinal fluid and MRI findings, as well as brain pathology. Both the age at onset and the disease course varied markedly among cases and did not show peculiarities associated with the V203I genotype (Table 1).

The extensive analyses published by Vallabh Minikel and co-workers [2] allowed us to address the issue of penetrance of the V203I variant in the light of the human genome variation. They counted 17 alleles (from 16 individuals, 1 homozygous) in the cohort of CJD patients (n = 16025) and 3 alleles from the EcAC cohort (n = 60706). A crude case–control analysis would result in OR = 20.20 (95% confidence interval: 5.78–108.20; p < 0.0001) associated to the presence of the 203I allele. Subsequently to this study, two additional heterozygous V203I patients were reported [6,8], plus the case here described.

In line with the conclusion drawn in the aforementioned study [2], the present review of cases does not support the high penetrance Mendelian model. It is worth noting that one patient homozygous for the V203I variant showed a late onset (73 years) and a relatively prolonged duration of disease (2 years). Conversely, the recurrent description of cases from diverse ancestries, the presence of the V203I variant in healthy individuals and the lack of segregation in families support the hypothesis that V203I variant is a risk genotype for CJD. The differential distribution of allele frequencies between the patient series and the reference population is consistent with such hypothesis. This interpretation warrants further analyses to be supported. However, the assumption that V203I may exert a non-Mendelian effect should be carefully considered in the clinical scenario.

Once a rare germline variant associated with a devastating disease is discovered in a family, the concern about recurrence is overwhelming, as the mutation accounts for a recurrence risk of up to 50% in first-degree relatives. Interpreting the relevant genetic variant as a risk factor, and not as a Mendelian mutation, implies that the recurrence profile should not be drawn according to the autosomal dominant model, which underlies the familial segregation of typical gCJD. A genetic risk factor with moderate effect size causes an increased liability to develop the disease with respect to the general population, which in turn is very low in a rare disorder such as CJD. This information is pivotal in the context of genetic counselling for families carrying a genetic risk variant. This is the case, for instance, of the *ApoE* ε4 allele, which confers an increased risk for Alzheimer’s disease but is far from being considered a Mendelian mutation, since multiple genetic variants, as well as non-genetic determinants, jointly modify the risk of sporadic Alzheimer’s disease [12]. All the involved families should be offered an appropriate genetic counselling procedure, in order to convey knowledge-based information and allow family members to understand the facts and the available options.

## Data Availability

There is no data set associated with the article.

## Geolocation information

The study was based in Genoa, Italy.
